# Description of ‘Desulfobacterium corrodens’ strain IS4 as Desulfosediminibacter corrodens gen. nov. sp. nov. and reclassification of Desulfopila inferna as Desulfosediminibacter infernus comb. nov.

**DOI:** 10.1099/ijsem.0.007342

**Published:** 2026-10-30

**Authors:** Stefan Dyksma, Maximilian von Frieling, Dominik Stickel, Jacqueline Wolf, Meina Neumann-Schaal, Cathrin Spröer, Boyke Bunk, Mathias Müsken, Michael Pester

**Affiliations:** 1 Chair of Microbial Physiology, Technical University of Munich, Freising, Germany; 2 Department of Microorganisms, Leibniz Institute DSMZ – German Collection of Microorganisms and Cell Cultures, Braunschweig, Germany; 3 Leibniz Institute DSMZ – German Collection of Microorganisms and Cell Cultures, Chemical Analytics and Metabolomics, Braunschweig, Germany; 4 Braunschweig Integrated Centre of Systems Biology (BRICS), Rebenring 56, D-38106 Braunschweig, Germany; 5 Department of Bioinformatics, Leibniz Institute DSMZ – German Collection of Microorganisms and Cell Cultures, Braunschweig, Germany; 6 Central Facility for Microscopy, Helmholtz Centre for Infection Research, Inhoffenstraße, 738124 Braunschweig, Germany

**Keywords:** microbiologically influenced corrosion, sulphate reduction, sulphur cycle

## Abstract

Sulphate-reducing bacteria of the families *Desulfobulbaceae* and *Desulfocapsaceae* are ubiquitously distributed and abundant in marine sediments. The present study characterizes strain IS4^T^, a sulphate reducer that was formerly isolated from a coastal sediment. Phylogenetic analyses based on 16S rRNA gene and marker protein sequences identified *Desulfopila inferna* JS_SRB250Lac^T^ as the closest relative of strain IS4^T^, which is, however, polyphyletic with the type species *Desulfopila aestuarii* MSL86^T^. Both phylogenetic analyses and whole-genome comparisons suggest that strains IS4^T^ and JS_SRB250Lac^T^ represent different species of a new genus within the family *Desulfocapsaceae*. Strains IS4^T^ and JS_SRB250Lac^T^ further differ from *D. aestuarii* MSL86^T^ by their capacity to grow autotrophically, the presence of cyclic fatty acids (C_17 : 0_ cyclo *ω*7c) and their polar lipids that can include phosphatidylcholine, lysophosphatidylcholine, phosphatidylglycerol and phosphatidylethanolamine, while phosphatidylserine was absent. During lithoautotrophic growth of strain IS4^T^ with metallic iron as an electron donor, molecular hydrogen accumulated to a higher level than it was produced abiotically, suggesting that corrosion potential is a distinguishing feature between strains IS4^T^ and JS_SRB250Lac^T^. On the basis of phenotypic, phylogenetic and genomic characteristics, we propose the novel species and genus *Desulfosediminibacter corrodens* gen. nov. sp. nov. The type strain is IS4^T^ (=DSM 15630^T^=JCM 39320^T^). In addition, we suggest the reclassification of *D. inferna* as *Desulfosediminibacter infernus* comb. nov.

## Introduction

In anoxic environments, micro-organisms can gain energy from dissimilatory sulphate reduction, a metabolic trait that unfolds in representatives across 19 bacterial and 2 archaeal phyla [1]. This globally important process accounts for one-third of organic matter mineralization in the seabed and can even account for up to 50% of carbon mineralization in coastal sediments [2, 3]. Members of the families *Desulfobacteraceae*, *Desulfobulbaceae* and *Desulfocapsaceae* are ubiquitously distributed and often among the most abundant sulphate-reducing bacteria (SRB) in coastal sediments [4, 5].

SRB not only provide important ecosystem services in ocean environments, such as recycling organic matter, but are also associated with processes of enormous economic consequences like oil and gas souring and microbiologically influenced corrosion (MIC) [6, 7]. In the context of MIC, Dinh *et al*. enriched and isolated SRB from a coastal sediment on metallic iron (Fe^0^) as an electron donor [8]. They described the property of their isolate ‘Desulfobacterium corrodens’ strain IS4^T^ to directly take up electrons from Fe^0^ and thereby provided evidence for a novel mode of MIC [8]. In contrast, stainless steel did not serve as an electron donor for strain IS4^T^, suggesting that it is unable to extract electrons directly from Fe⁰ but rather relies on the formation of molecular hydrogen (H_2_) as an electron carrier for Fe⁰ oxidation [9].

Strain IS4^T^ has never been validly published. Some more characteristics of this strain were described later, as it is a close phylogenetic relative of *Desulfopila inferna* JS_SRB250Lac^T^ [10] and more distantly related to *Desulfopila aestuarii* MSL86^T^ [11], two SRB isolated from coastal and estuarine sediments, respectively. The present study characterizes the phylogeny, physiology and chemotaxonomy of the sulphate-reducing strain IS4^T^, for which we propose the name *Desulfosediminibacter corrodens* gen. nov. sp. nov., representing the type species of the new genus. Phylogenetic and whole-genome comparisons clearly separate strain IS4^T^ and *D. inferna* JS_SRB250Lac^T^ from the type species of the genus *Desulfopila*, *D. aestuarii* MSL86^T^. Thus, we further suggest reclassification of *D. inferna* to *Desulfosediminibacter infernus* comb. nov.

## Phylogeny and genomic analysis

DNA extraction and genome sequencing on a PacBio Sequel IIe system (Pacific Biosciences) was performed as described recently [12]. Long-read genome assembly was performed with the Microbial Assembly protocol included in SMRTlink version 10.2 using a target genome size of 6 Mbp. The genome size of strain IS4^T^ was estimated to be 5,560,733 bp with a G+C content of 48.1 mol% (Table S1, available in the online Supplementary Material). A total of 53 tRNAs and 5,688 protein-coding genes were predicted with RAST [13]. Three rRNA operons were identified in the genome of strain IS4^T^, i.e. three gene copies each that encode the 5S, 16S and 23S rRNA gene. Sequence similarities of its 16S rRNA genes are 99.7–100.0%. The 16S rRNA gene sequence that is represented by two identical copies found in the genome was used for phylogenetic reconstruction (Fig. 1). Sequences were aligned with MAFFT (E-INS-i) [14], and tree calculation was performed with IQ-TREE 2 (version 2.3.6) using ultrafast bootstraps (*n*=1,000) after automatic substitution model selection (GTR+F+R4) [15]. The 16S rRNA gene of IS4^T^ shared 96.8% sequence identity with its closest relative *D. inferna* JS_SRB250Lac^T^ and 94.2% identity with *D. aestuarii* MSL86^T^, which is below the recommended threshold (94.5%) to separate genera by 16S rRNA gene similarities [16]. Strain IS4^T^ also shared 95% similarity with *Desulfocastanea catecholica* DSM 3882^T^; however, the phylogenetic reconstruction revealed that strain IS4^T^ and *D. inferna* form a separate cluster apart from both *D. catecholica* and *D. aestuarii* (Fig. 1). This was supported by analysis of amino acid sequences of the dissimilatory sulphite reductase (DsrAB) and adenosine 5′-phosphosulphate reductase (AprAB) (Fig. S1) as hallmark enzymes of the sulphate reduction pathway. To further confirm the placement of strain IS4^T^, a phylogenomic tree was constructed based on 21,852 variable amino acid positions of 120 single-copy marker genes extracted and aligned with GTDB-Tk [17]. Tree calculation was performed with IQ-TREE 2 using ultrafast bootstraps (*n*=1,000) after automatic substitution model selection (LG+F+I+R6). The phylogenomic approach again identified *D. inferna* as a neighbour of strain IS4^T^ (Fig. 2), whereas *D. aestuarii* was consistently placed closer to the genus *Desulfosediminicola* (Figs 1, 2 and S1). Strain IS4^T^ shared genome-wide average nucleotide identity (ANI) [18] of 73.1% with *D. inferna* JS_SRB250Lac^T^ and 68.6% with *D. aestuarii* MSL86^T^, indicating that strains IS4^T^ and JS_SRB250Lac^T^ represent distinct species of the same genus [19]. In addition, an average amino acid identity (AAI) matrix was calculated for the 34 genomes of the families *Desulfobulbaceae* and *Desulfocapsaceae* used for phylogenomic analysis (Fig. S2). Similarly, the AAI value between strain IS4^T^ and *D. inferna* (74.5%) confirms their affiliation with the same genus, whereas the AAI between strain IS4^T^ and the type species of the genus *Desulfopila*, *D. aestuarii* (64.1%), was close to the proposed threshold for genus delineation within the family *Desulfocapsaceae* (61.5±1.5%, mean±sd, *n*=4) [20]. *D. catecholica* DSM 3882^T^ shared highest AAI values with the highest AAI values with *Desulfomarina profundi* DSM 111364^T^ (69%) and *Desulforhopalus singaporensis* DSM 12130^T^ (68.7%), while the AAI value between *D. catecholica* and strain IS4^T^ is 64.6%. Furthermore, the percentage of conserved proteins supports the conclusion that strain IS4^T^ and *D. inferna* are distinct species of a new genus (Fig. S3).

**Fig. 1.**
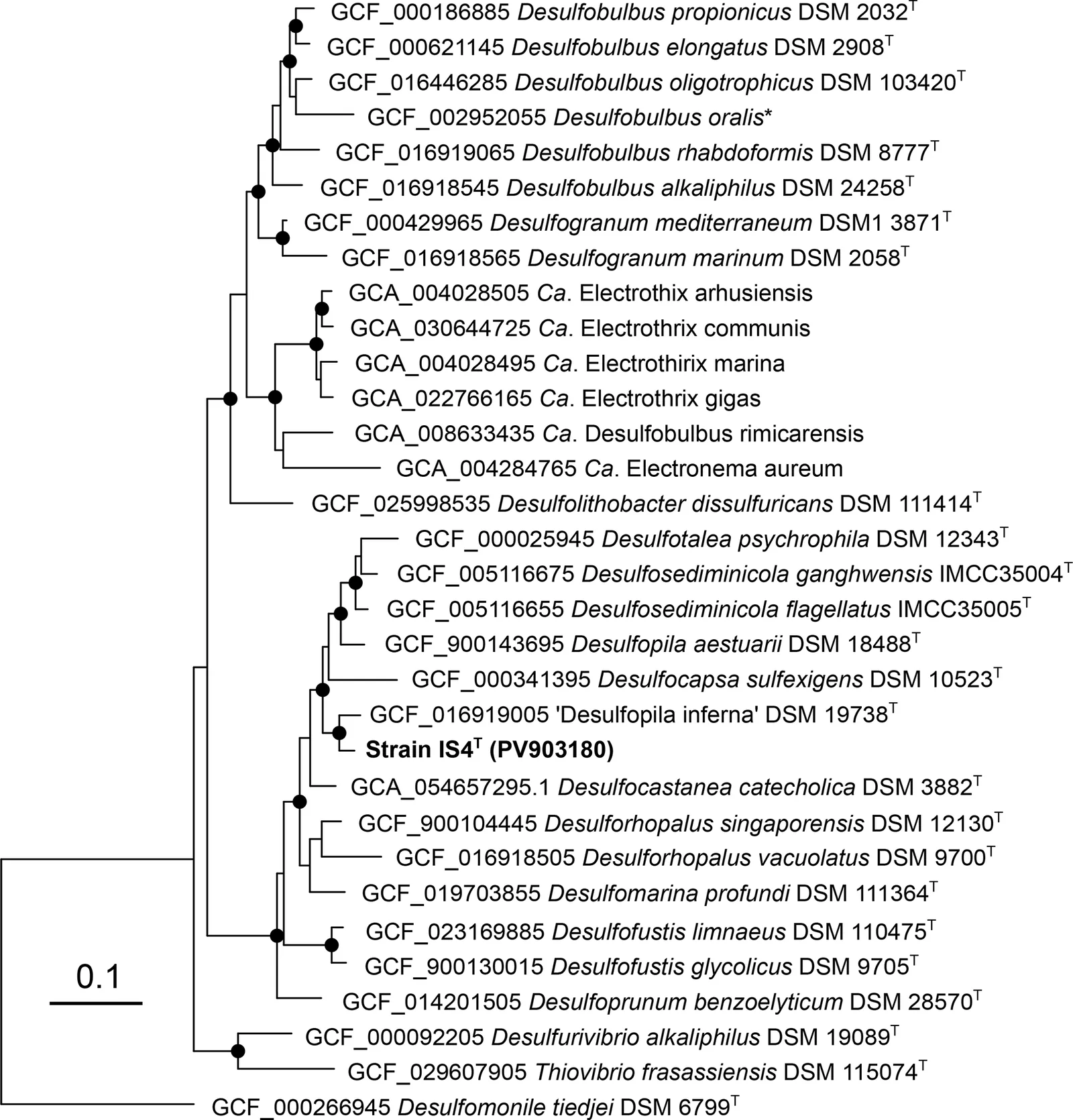
Maximum-likelihood phylogenetic tree based on 16S rRNA gene sequences of strain IS4^T^ and related strains. The 16S rRNA gene sequence of *Desulfomonile tiedjei* DSM 6799^T^ was used as an outgroup. Black dots denote bootstrap support ≥90%. The scale bar represents 5% sequence divergence.

**Fig. 2.**
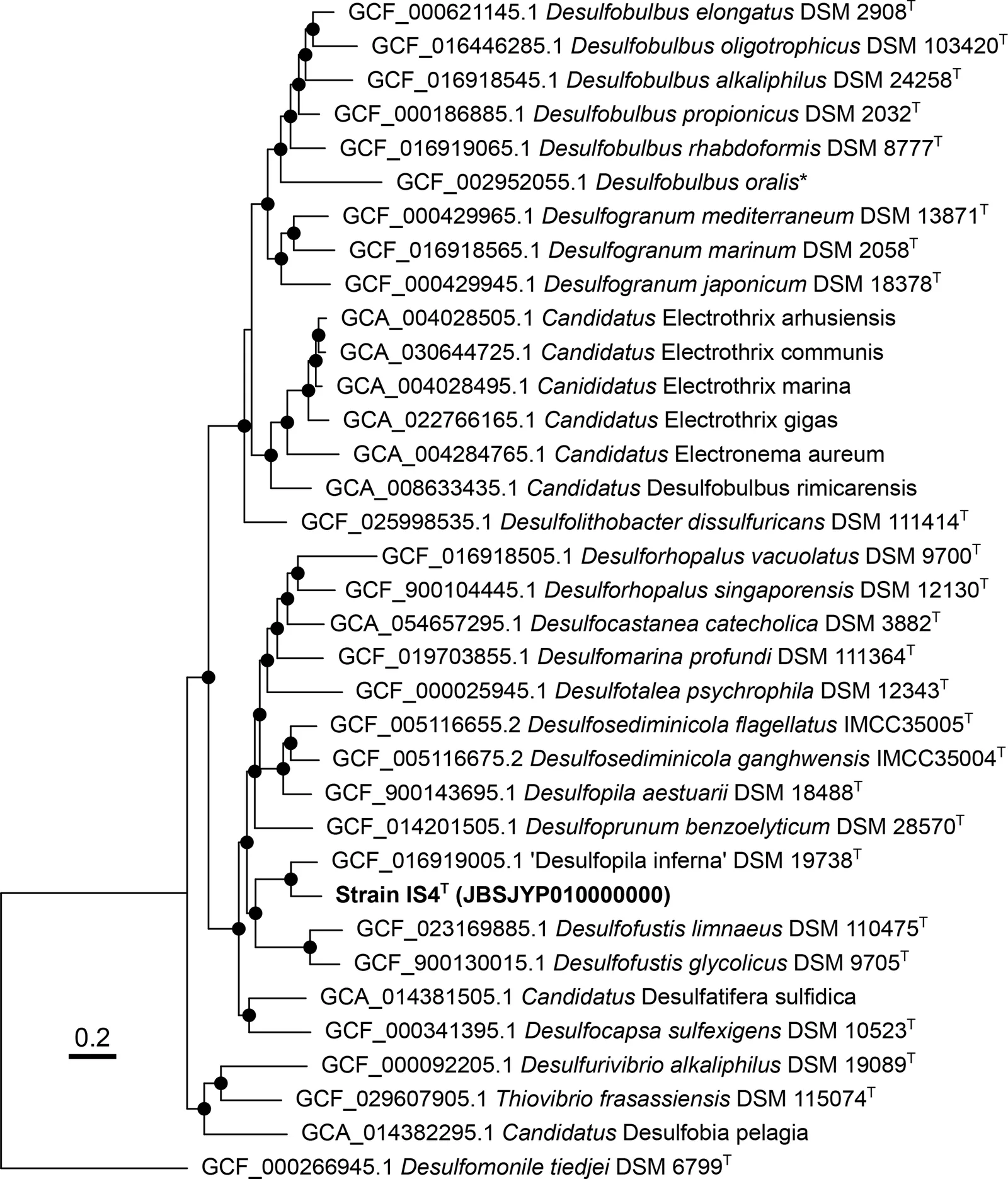
Maximum-likelihood phylogenomic tree based on a concatenated alignment of 120 single-copy bacterial marker proteins (21,852 variable positions). *Desulfomonile tiedjei* DSM 6799^T^ was used as an outgroup. Black dots denote bootstrap support ≥90%. The scale bar represents 10% sequence divergence.

The genomes of strains IS4^T^, JS_SRB250Lac^T^ and MSL86^T^ were annotated with RAST [13] and eggNOG-mapper version 2 [21]. Genomic features that were shared by these SRB are the potential for fatty acid degradation (CoA transferase, acyl-CoA dehydrogenases, enoyl-CoA hydratase, 3-hydroxybutyryl-CoA dehydrogenase and acetyl-CoA acetyltransferase), lactate utilization (lactate permease and lactate utilization protein complex LutABC) and glycerol metabolism (glycerol transporter, glycerol kinase and glycerol-3-phosphate dehydrogenase). All three genomes further contain the complete canonical sulphate reduction pathway (*sat*, *aprAB-qmoABC* and *dsrABCDMKJOP*) as well as all genes of the Wood–Ljungdahl pathway (WLP). The WLP fulfils multiple functions in anaerobic bacteria, e.g. providing an electron sink for redox balancing in acetogens [22], allowing syntrophic acetate-oxidizing bacteria and SRB to utilize acetate as a carbon and energy source [23, 24], or as an important carbon fixation pathway [25]. An uptake hydrogenase (NiFe group 1) was also identified in the genomes of strains IS4^T^, JS_SRB250Lac^T^ and MSL86^T^, indicating that the WLP might be used for autotrophic growth as demonstrated earlier by enzyme activity assays with *D. inferna* JS_SRB250Lac^T^ [10].

## Morphological characterization and physiology

Morphology and motility were observed using phase contrast microscopy and scanning electron microscopy (SEM) as described recently with slight modification [26]. Samples for SEM were fixed in a stepwise process directly in the growth medium by addition of glutaraldehyde (2% final) for 30 min, followed by the addition of formaldehyde for another 30 min. In addition, a CPD300 (Leica Microsystems) was used for automated critical point drying. Cells appeared as rods, 2.0–5.0 µm long and 0.3–0.5 µm wide (Fig. 3). Cells were not motile.

**Fig. 3.**
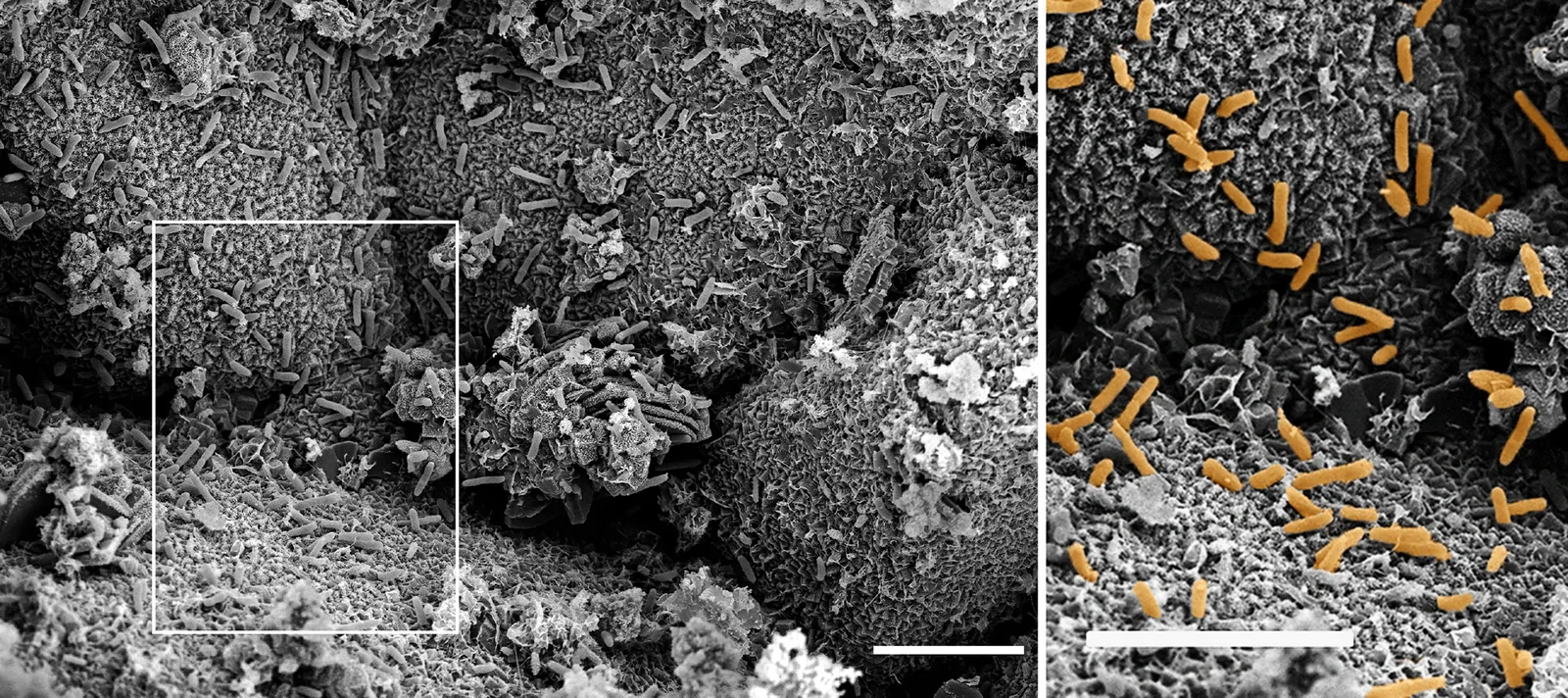
SEM image of strain IS4^T^ cultivated with metallic iron as an electron donor. Bacteria are colorized for better visualization in the enlarged section of the image. The scale bar represents 10 µm.

Strain IS4^T^ as well as the reference strains JS_SRB250Lac^T^ and MSL86^T^ were obtained from DSMZ (German Collection of Microorganisms and Cell Cultures). All growth experiments were performed in triplicate in 25-ml culture tubes with a marine (2% NaCl) mineral medium [27] supplemented with trace elements SL10 [28], seven-vitamin solution [27] and selenite–tungstate solution [29]. Strains were grown under strictly anoxic conditions in defined bicarbonate-buffered, sulphide-reduced media as described earlier [27]. The final pH was adjusted to 7.0 to 7.45. If not otherwise stated, growth was determined by following optical density (measured at 600 nm) over time. To test for temperature, pH and NaCl optimum the medium was supplemented with 10 mM lactate as an electron donor and carbon source and 10 mM sulphate as an electron acceptor. Growth was tested in the range 4–45 °C. The optimum growth temperature of strain IS4^T^ (28 °C) was subsequently selected for testing pH and NaCl optimum (Table 1). The effect of NaCl concentration and pH on growth of strain IS4^T^ was tested in the range 0–5% (steps of 0.5%) and pH 5.5–9.0 (steps of 0.5), respectively. Strain IS4^T^ grew at all tested salinities with an optimum between 0.5 and 3% NaCl and between pH 6.5 and 9.0 (Table 1). The optimum pH for growth was pH 7.5–8.5, while no growth occurred at pH 6 and below. The closest phylogenetic relative, strain JS_SRB250Lac^T^, and *D. aestuarii* MSL86^T^ were included as reference strains in the growth experiments (Table 1). Growth of strain MSL86^T^ was determined by measuring the produced biomass after 3 weeks of incubation as the cells aggregated during growth [11]. All three strains have comparable optima for temperature, salinity and pH (Table 1).

**Table 1.** Comparison of the major characteristics that differentiate strain IS4^T^ (1) from its closest phylogenetic relative *D. inferna* JS_SRB250Lac^T^ (2) and from *D. aestuarii* MSL86^T^ (3) representing the type species of the genus *Desulfopila* Data were obtained from Gittel *et al*. [10] for strain JS_SRB250Lac^T^ and from Suzuki *et al*. [11] for strain MSL86^T^ unless otherwise indicated.

Characteristic	1	2†	3‡
Type strain	IS4^T^	JS_SRB250Lac^T^	MSL86^T^
Isolation source	Coastal sediment*	Tidal sediment from 2.5 m depth	Estuarine sediment
Cell size (µm)	2.0–5.0×0.3–0.5	1.0–2.0×0.3–0.5	1.9–3.8×0.7–1.2
Motility	−	−	+
Temperature range, optimum (°C)	15–35, 28–30	10–35, 28	10–40, 35
pH range, optimum	6.5–9.0, 7.5–8.5	6.5–8.5, 7.0§	6.3–8.5, 7.5–7.6
NaCl range, optimum (%)	0–5.0, 0.5–3.0	0.5–5.0, 2.0–3.0	0–5, 1
Electron donors with sulphate			
H_2_/CO_2_	+	+	−†
H_2_+acetate	+	+	−
Lactate	+	+	+
Pyruvate	+	+	+
Malate	−	−	−
Succinate	−	+	−
Fumarate	−	+	+
Benzoate	+	−	+§
Formate	+	+	+
Acetate	−	−	−
Propionate	−	−	−
Butyrate	−	+	−
Valerate	na	+	na
Caprate	na	+	na
Caproate	na	+	na
Betaine	na	−	na
Choline	na	−	na
Glycine	−	−	−
Serine	(+)	−	−
Proline	+	+	+§
Alanine	−	−	−
Glutamate	−	-§	−
Aspartate	(+)	-§	−
Methanol	−	-§	−
Ethanol	(+)	+	+
1-Propanol	+	+	+
1-Butanol	−	+	+
Glycerol	+	+	+
Sorbitol	na	−	na
Mannitol	na	−	na
Electron acceptors with lactate†, ‡ or H_2_§			
Sulphate	+	+	+
Thiosulphate	+	−	+
Sulphite	+	+	+
Fumarate	-	na	+
Fe(III)	+	+§	+§
Nitrate	−	−	na
Fermentation in the absence of sulphate			
Lactate	+	−	−
Pyruvate	−	+	+
Fumarate	+	+	+
Malate	−	−	−

*Dinh *et al*., 2004 [8].

†Gittel *et al*., 2010 [10].

‡Suzuki *et al*., 2007 [11].

§This study.

(+), weak growth; +, growth; −, no growth; NA, not tested or data not available.

The following electron donors were tested with sulphate (10 mM) as an electron acceptor: H_2_, H_2_ with acetate, lactate, pyruvate, benzoate, malate, fumarate, succinate, formate, acetate, propionate, butyrate, glycine, alanine, serine, proline, aspartate, glutamate, methanol, ethanol, propanol, butanol and glycerol. All organic substrates were added to a final concentration of 10 mM. Several organic acids, amino acids and alcohols supported growth of strain IS4^T^ under sulphate-reducing conditions (Table 1). Butanol was not used as a substrate, which was supported by the genomic analysis. NADH-dependent butanol dehydrogenases that potentially catalyse the initial step of butanol degradation were only found in the genomes of strains JS_SRB250Lac^T^ and MSL86^T^. Only two genes encoding alcohol dehydrogenases (EC 1.1.1.1) were found in the genome of strain IS4^T^, whereas strains JS_SRB250Lac^T^ and MSL86^T^ encode six and nine different alcohol dehydrogenase proteins, respectively. No growth of strain IS4^T^ was observed with malate, succinate, glycine, alanine, glutamate and methanol as well as with the short-chain fatty acids acetate, propionate and butyrate. Autotrophic growth with H_2_/CO_2_ without acetate was possible. Strain IS4^T^ fermented lactate and fumarate. Hydrogen was used as an electron donor to test growth with the following alternative electron acceptors: thiosulphate (10 mM), sulphite (5 mM), nitrate (10 mM), fumarate (10 mM) and Fe(III) provided as ferrihydrite (10 mM). Fe(II) formation from Fe(III) during growth with ferrihydrite as an electron acceptor was followed by using the ferrozine assay [30]. Strain IS4^T^ was able to use Fe(III), sulphite and thiosulphate as electron acceptors but was unable to reduce nitrate and fumarate. The products of fermentation and sulphate respiration were analysed using ion chromatography as follows. Anions were separated in a SykroGel AX300 column (Sykam, Germany) using 5 mM NaCO_3_ as eluent. Organic acids were separated in a SykroGel EX450 column (Sykam, Germany) using 7% acetonitrile and 0.7 mM perfluorobutanoic acid as eluent. Both anions and organic acids were quantified using a conductivity detector.

## Growth with metallic iron (Fe^0^) as an electron donor

All three strains were tested for autotrophic growth with metallic iron (Fe^0^) as an electron donor. The medium without a reducing agent was supplemented with 20 g iron granules (size 1–2 mm, 99.98% Fe) per 100 ml. Incubations were performed in triplicate in 250-ml culture bottles filled with 100 ml medium. Under anoxic conditions, protons from water (H^+^) or the end product of sulphate reduction, hydrogen sulphide (H_2_S), can react abiotically with Fe^0^ and form H_2_ [31]. Abiotically produced H_2_ in sterile controls steadily increased to 9.2 mM H_2_ at day 37 (Fig. 4). H_2_ also accumulated in cultures of *D. aestuarii* MSL86^T^, while the sulphate concentration only decreased by 0.6 mM, confirming that this strain is not able to grow autotrophically with H_2_ as an electron donor [11]. Abiotically produced H_2_ was not observed in cultures of *D. inferna* JS_SRB250Lac^T^ (Fig. 4). Strain JS_SRB250Lac^T^ consumed 2.7 mM sulphate, which can be well explained by the complete oxidation of abiotically produced H_2_ (4 mole of H_2_ are required for the reduction of 1 mole sulphate). In contrast, H_2_ accumulated to high levels (maximum of 21.3 mM) in cultures of *D. corrodens* IS4^T^ (Fig. 4), indicating that metallic iron was corroded more efficiently by strain IS4^T^ compared to the abiotic process [8]. A black corrosion crust [7, 8] on the metallic substrate was only observed in cultures of *D. corrodens* IS4^T^.

**Fig. 4.**
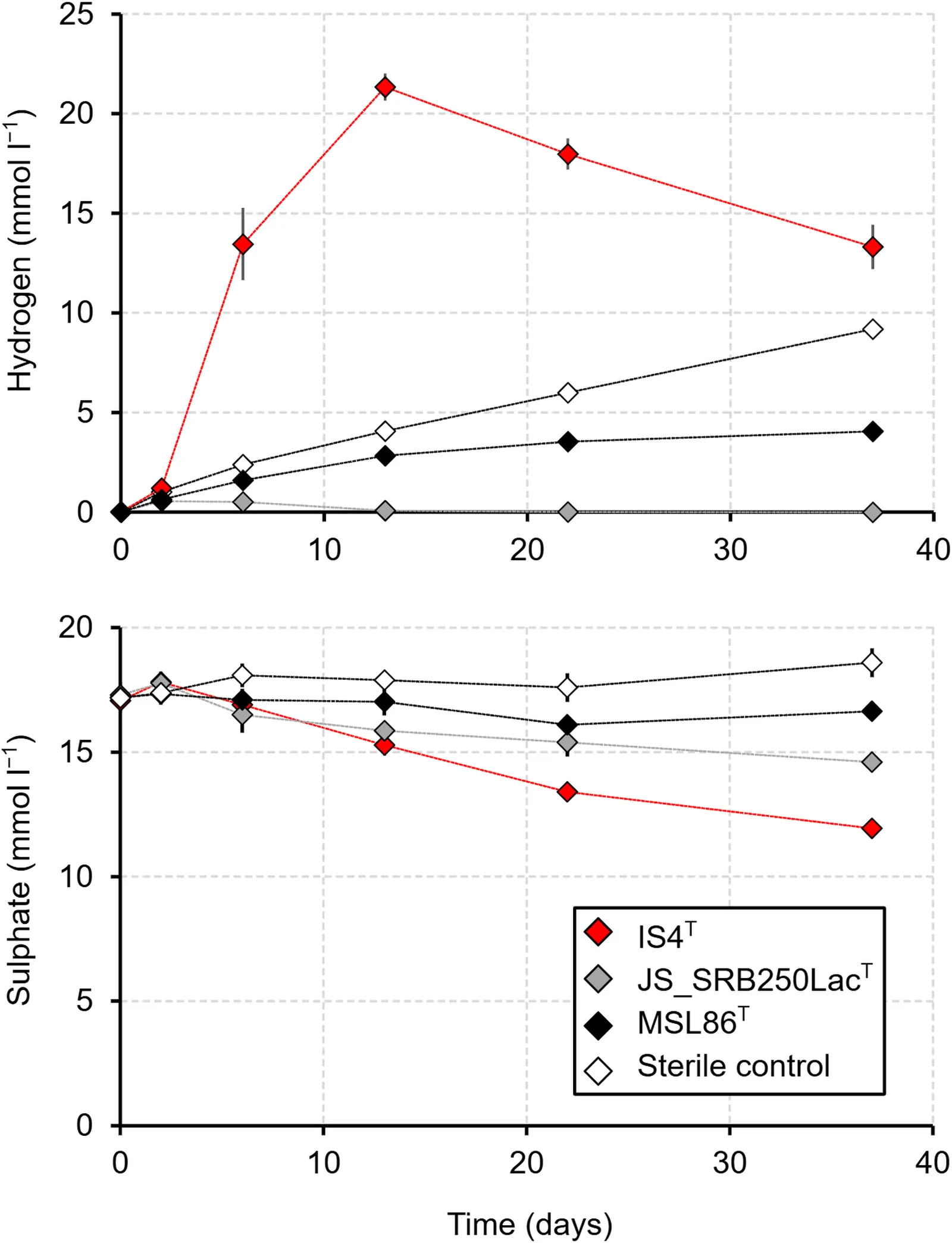
Incubation with metallic iron as sole electron donor. Hydrogen formation (top) and sulphate consumption (bottom) in cultures of strains IS4^T^, JS_SRB250Lac^T^ and MSL86^T^ as well as in sterile control incubations.

## Chemotaxonomy

Cellular fatty acids, respiratory quinones and polar lipids of the strains IS4^T^, JS_SRB250Lac^T^ and MSL86^T^ were analysed after cultivation at 28 °C on the mineral medium used for growth experiments supplemented with lactate. Cellular fatty acids were analysed after conversion to fatty acid methyl esters (FAMEs), following the protocol described by Sasser for saponification, methylation and extraction [32]. FAMEs were analysed by GC coupled to a flame ionization detector using the Sherlock Microbial Identification System (MIDI, version 6.1, TSBA6 database) and subsequently analysed by GC-MS on an Agilent GC-MS 7000D system to validate the identity of all fatty acids [33]. To determine the double-bond position of monounsaturated fatty acids, FAMEs were additionally derivatized to dimethyl disulphide adducts followed by GC-MS analysis [34]. Similar to the closest phylogenetic neighbour *D. inferna*, major fatty acids that constitute more than 5% of the total fatty acids of strain IS4^T^ were C_16 : 0_ (14.6%), C_16 : 1_ *ω*7c (18.0%), C_16 : 1_ *ω*5c (24.8%), C_17 : 1_ *ω*6c (6.6%) and C_18 : 1_ *ω*7c (15.2%). The presence of cyclic fatty acids (C_17 : 0_ cyclo *ω*7c) discriminates the strains IS4^T^ and JS_SRB250Lac^T^ from *D. aestuarii* MSL86^T^ (Table S2), which has been reported earlier [10, 35].

Respiratory quinones were extracted from wet biomass and analysed by HPLC coupled to a diode array detector and a high-resolution mass spectrometer [33]. MK-5 (H2) was the major quinone of strain IS4^T^. The major menaquinone MK-8 (H4) that was previously reported for *D. aestuarii* [11] could not be confirmed. All three strains IS4^T^, JS_SRB250Lac^T^ and MSL86^T^ contained MK-5 (H2) as a major menaquinone. However, *D. inferna* JS_SRB250Lac^T^ additionally contained substantial amounts of ubiquinone Q-5 (Fig. S4), which is not common for SRB [36].

Polar lipids were extracted from wet biomass following a modified Bligh and Dyer extraction protocol [37]. Samples were extracted twice with methanol:dichloromethane (DCM): 0.3% NaCl (2 : 1 : 0.8, v/v/v) by sonication at ambient temperature for 10 min. The combined DCM phases were additionally extracted twice with 0.3% NaCl and subsequently evaporated under a stream of nitrogen. Dried extracts were resolved in hexane:isopropanol:water (718 : 271 : 10 v/v/v) and filtered through a regenerated cellulose syringe filter (Minisart, RC4, Sartorius). Polar lipid extracts were analysed by HPLC-MS on an Agilent 1290 Infinity II LC system, coupled to a high-resolution 6545-Q-ToF mass spectrometer (Agilent Technologies) as described previously [38]. Membrane lipids that were identified in all three strains are phosphatidylethanolamine (PE) and diphosphatidylglycerol (DPG). The closely related strains IS4^T^ and JS_SRB250Lac^T^ additionally had either phosphatidylcholine (PC) and lysophosphatidylcholine (LPC) or phosphatidylglycerol and lysophosphatidylethanolamine, respectively. Phosphatidylserine was only detected in strain MSL86^T^. A detailed polar lipid composition of strains IS4^T^, JS_SRB250Lac^T^ and MSL86^T^ is provided as supplementary material (Table S3).

## Conclusion

Phylogenetic analysis consistently showed a close relationship between *D. corrodens* IS4^T^ and *D. inferna* JS_SRB250Lac^T^ and that they are more distantly related to *D. aestuarii* MSL86^T^ (Figs 1, 2 and S1). Although the strains IS4^T^ and JS_SRB250Lac^T^ have distinct polar lipid compositions (Table S3), their shared ANI (73.1%) and AAI (74.5%) values argue against separation into two distinct genera. The capacity for autotrophic growth and presence of cyclic fatty acids further distinguish the newly proposed genus *Desulfosediminibacter* from *Desulfopila*. Strain IS4^T^ differs from JS_SRB250Lac^T^ by its ability to use benzoate with sulphate as an electron acceptor. Corrosion of metallic iron in addition to the mere consumption of abiotically formed H_2_ from the reaction of H^+^ and/or H_2_S with Fe was only observed with *D. corrodens* IS4^T^. The phylogenetic, physiological and chemotaxonomic results as well as the whole-genome analyses show that strains IS4^T^ and JS_SRB250Lac^T^ represent different species and belong to the new genus *Desulfosediminbacter*.

## Description of *Desulfosediminibacter* gen. nov.

*Desulfosediminibacter* (De.sul.fo.se.di.mi.ni.bac’ter. L. neut. n. *sedimen*, sediment; L. pref. de, from; L. neut. n. sulphur, sulphur; N.L. masc. n. *bacter*, rod or staff; N.L. masc. n. *Desulfosediminibacter*, a rod-shaped sulphate-reducing bacterium from sediments).

Strictly anaerobic, SRB. Cells are non-spore-forming, immotile rods. Mesophilic bacteria with optimal growth observed at pH 7.0–8.5. Predominant fatty acids are C_16 : 0_, C_16 : 1_ *ω*7c, C_16 : 1_ *ω*5c, C_17 : 1_ *ω*6c and C_18 : 1_ *ω*7c. Cyclic fatty acids (C_17 : 0_ cyclo *ω*7c) are present. MK-5 (H2) is the major respiratory menaquinone, but ubiquinone Q-5 was identified as well for *D. inferna*. Autotrophic growth is possible with H_2_ as an electron donor. The type species is *Desulfosediminibacter corrodens*.

## Description of *Desulfosediminibacter infernus* comb. nov.

*Desulfosediminibacter infernus* (in.fer′nus. L. masc. adj. *infernus*, that which is, or comes from, below, referring to the isolation of the type strain from a subsurface sediment).

Basonym: *Desulfopila inferna* Gittel *et al*. 2010.

The description of *Desulfosediminibacter infernus* comb. nov. is identical to the description of *Desulfopila inferna* by Gittel *et al*., 2010. In addition, the major respiratory menaquinone is MK-5 (H2) with substantial amounts of ubiquinone Q-5.

The type strain is JS_SRB250Lac^T^ (=DSM 19738^T^=NBRC 103921^T^).

## Description of *Desulfosediminibacter corrodens* sp. nov.

*Desulfosediminibacter corrodens* sp. nov. (cor.ro’dens. L. pres. part. *corrodens*, corroding, referring to its ability to corrode metallic iron).

Cells are immotile rods, 0.3–0.5 µm in width and 2.0–5.0 µm in length. The temperature range for growth is 15–35 °C, with an optimum at 28–30 °C. No growth occurred at 10 °C and 37 °C. Neutrophilic, with an optimum growth at pH 7.5–8.5. Growth is possible within a broad range of salt concentrations (0–5.0% NaCl). Sulphate reduction is observed with H_2_, lactate, pyruvate, benzoate, formate, proline, propanol and glycerol as an electron donor. Weak growth is further observed with serine, aspartate and ethanol. Strain IS4^T^ can utilize metallic iron as an electron donor. The following substrates are not used as electron donors: malate, succinate, glycine, alanine, glutamate, methanol, acetate, propionate and butyrate. Sulphate, sulphite, thiosulphate and Fe(III) provided as ferrihydrite are used as electron acceptors. Fermentation of lactate and fumarate is positive. Menaquinone MK-5 (H2) is the major respiratory quinone. Polar lipids are comprised of PE, DPG, PC and LPC.

The type strain IS4^T^ (=DSM 15630^T^=JCM 39320^T^)RB are the potential for fatty aci was isolated beforehand from a marine sediment collected near Wilhelmshaven, Germany [8]. Based on the genome sequence, the G+C content of the type strain is 48.1 mol%. The GenBank accession numbers for the 16S rRNA gene and the genome sequence of strain IS4^T^ are PV903180 and JBSJYP010000000, respectively.

## Supplementary material

10.1099/ijsem.0.007342 Supplementary Material 1.

## Abbreviations

AAI: average amino acid identity
ANI: average nucleotide identity
DCM: dichloromethane
DPG: diphosphatidylglycerol
FAMEs: fatty acid methyl esters
LPC: lysophosphatidylcholine
MIC: microbiologically influenced corrosion
PC: phosphatidylcholine
PE: phosphatidylethanolamine
SEM: scanning electron microscopy
SRB: sulphate-reducing bacteria
WLP: Wood–Ljungdahl pathway
