# Supplementary material for: Description of ‘Desulfobacterium corrodens’ strain IS4 as Desulfosediminibacter corrodens gen. nov. sp. nov. and reclassification of Desulfopila inferna as Desulfosediminibacter infernus comb. nov
Source: Int J Syst Evol Microbiol. 2026 Oct 30;76(10):007342. doi: 10.1099/ijsem.0.007342 (PMC13761340; doi:10.1099/ijsem.0.007342)
Supplement: Supplementary file 1 [file ijsem-76-07342-s001.pdf]

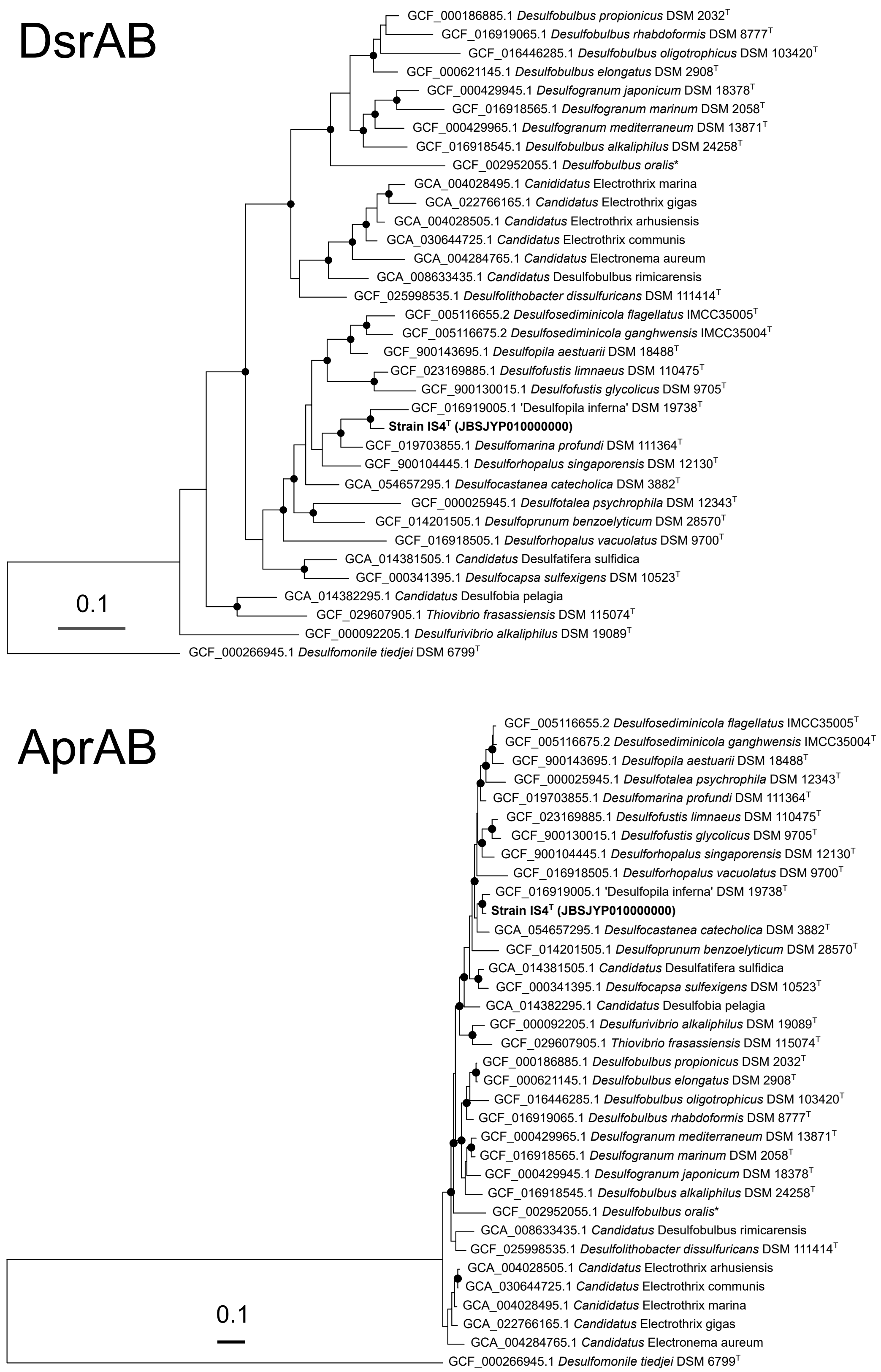

Supplementary Figure S1. Maximum likelihood phylogenetic tree based on dissimilatory sulphite reductase (DsrAB) and adenosine 5'-phosphosulphate reductase (AprAB) protein sequences. Black dots denote bootstrap support ≥ 90%. The scale bar represents 10% sequence divergence. AprAB and DsrAB trees were calculated with IQ-TREE 2 (version 2.3.6) using the substitution models LG+F+I+R3 and LG+I+G4, respectively.

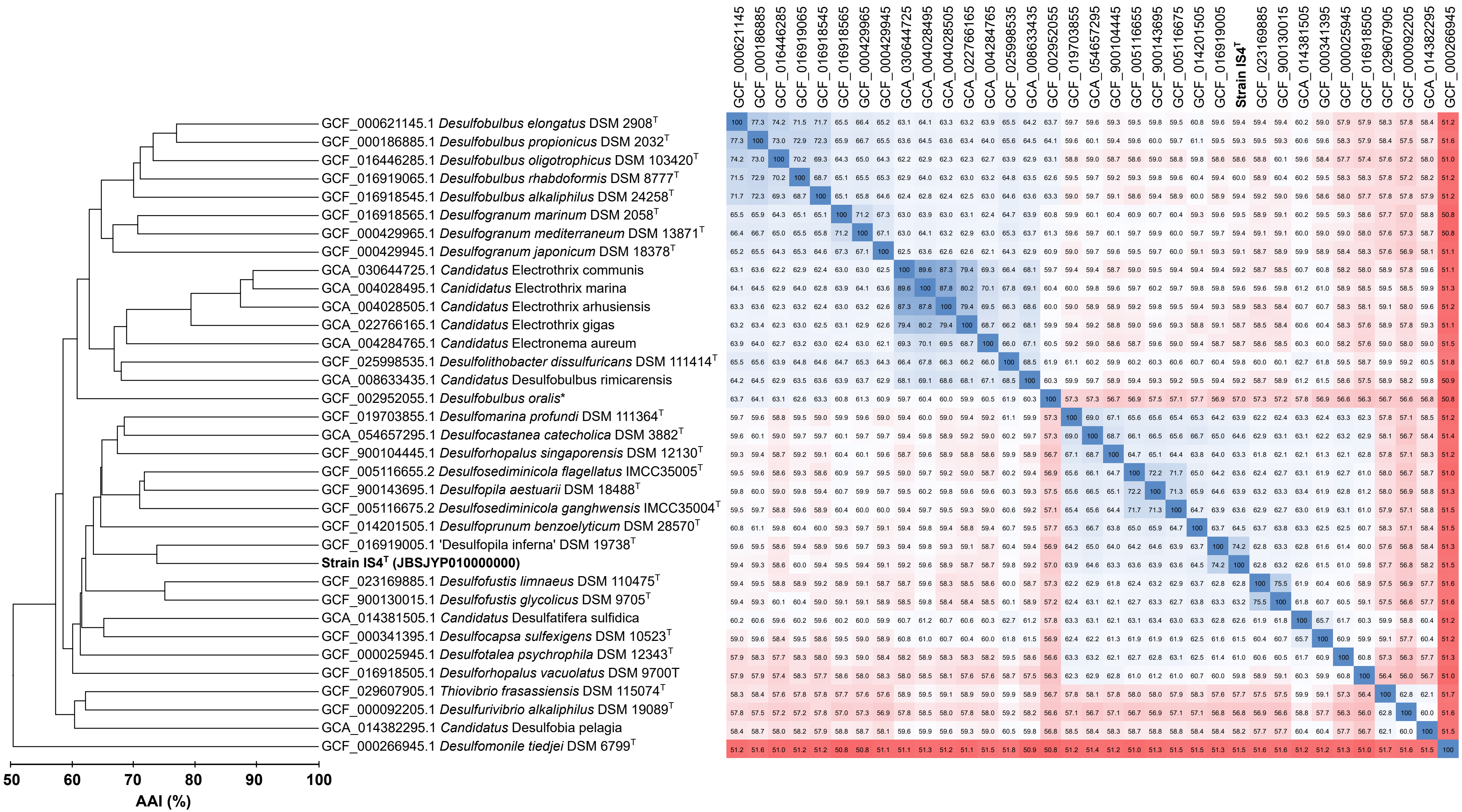

Supplementary Figure S2. UPGMA dendrogram and average amino acid identity matrix of selected genomes.

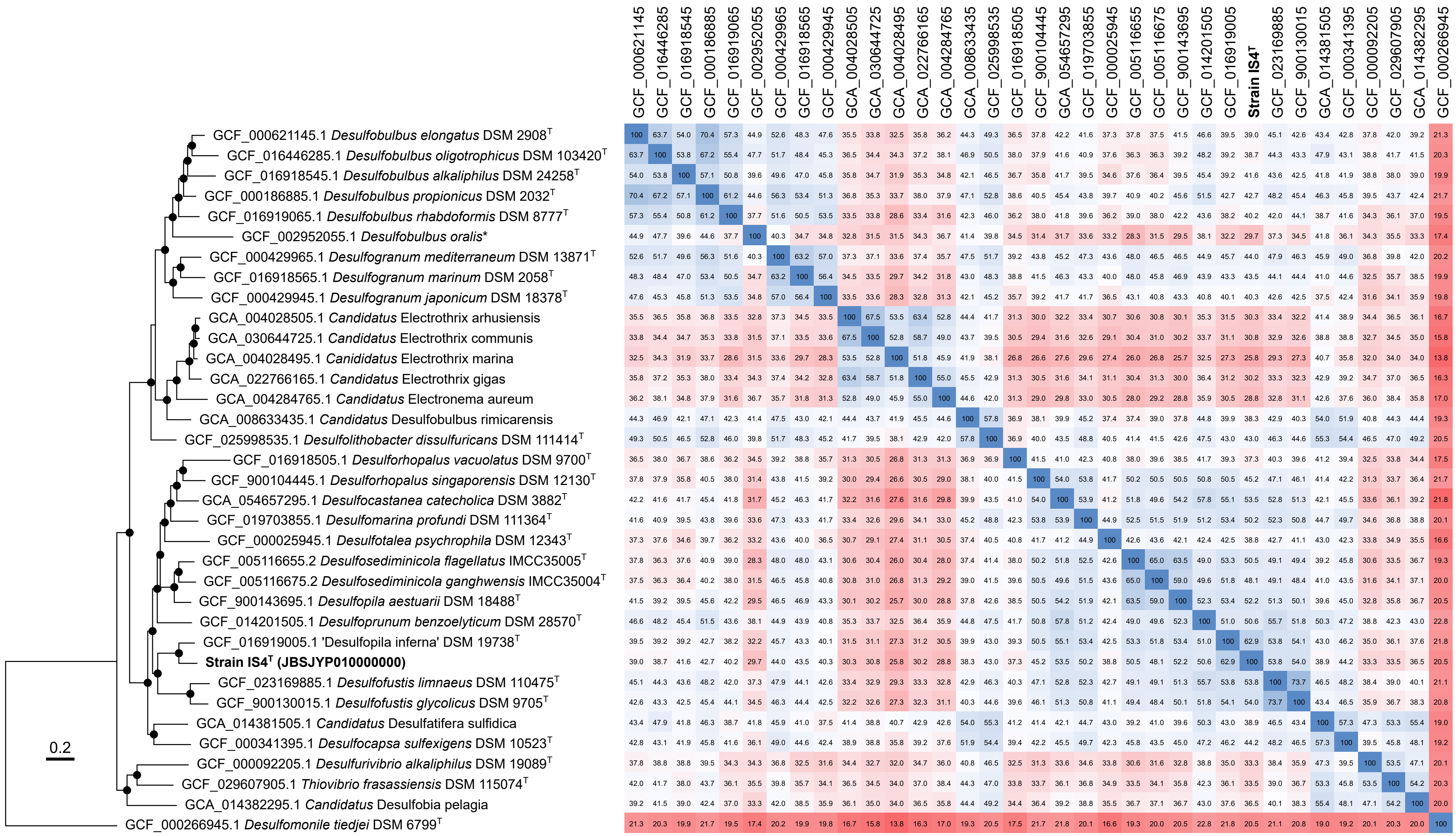

Supplementary Figure S3. Percentage of conserved proteins (POCP) of selected genomes. The order of the matrix corresponds to the phylogenogenomic tree (Figure 2).

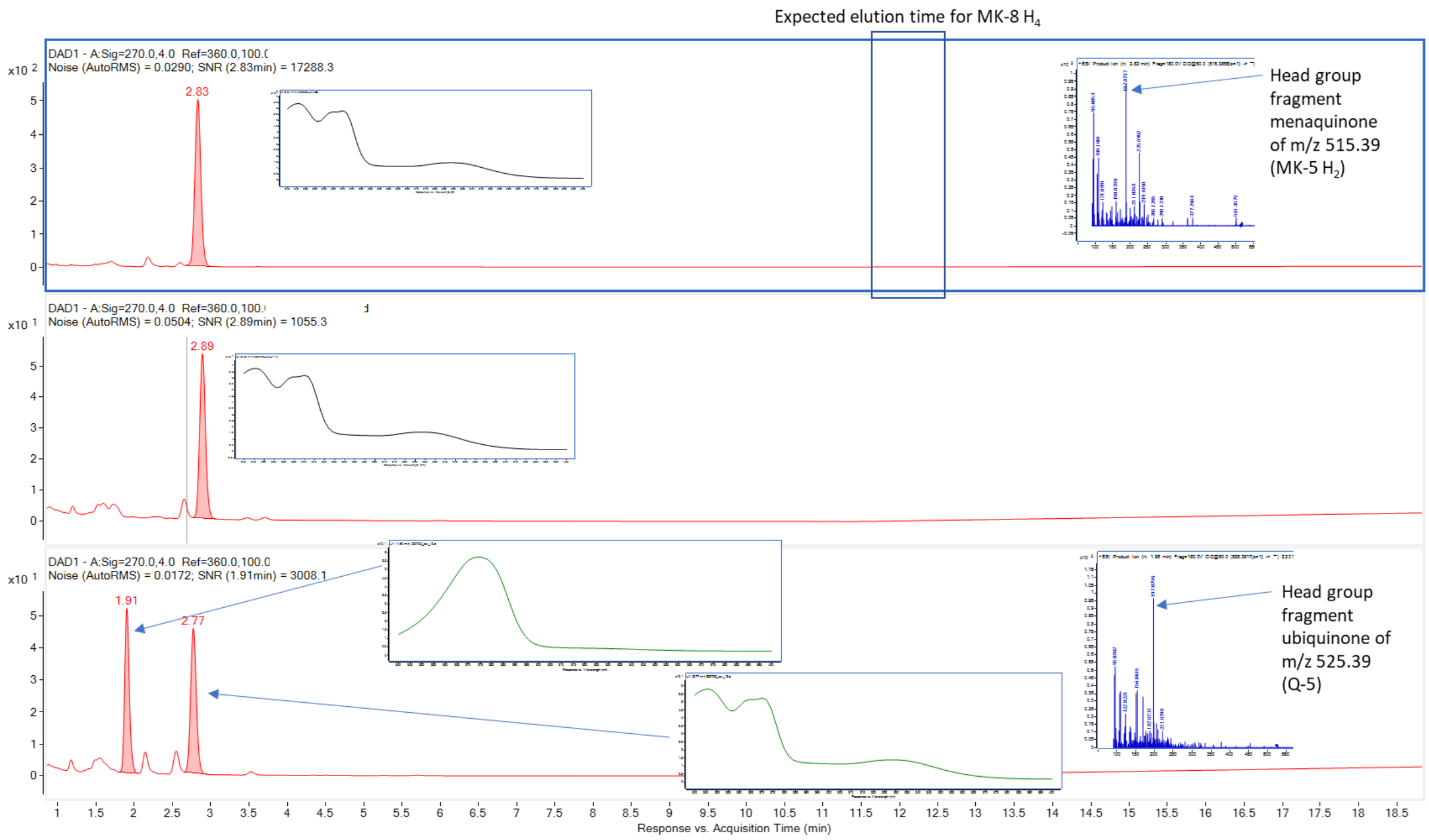

Supplementary Figure S4. Respiratory quinone analysis of strains IS4^T, MSL86^T and JS_SRB250Lac^T (top to bottom). Shown are the DAD signals at 270 nm and the inserted absorption spectra at 1.9 and 2.8 minutes, showing representative absorption spectra of ubiquinones and menaquinones, respectively. The insert in the top panel shows MS/MS fragmentation spectra of the menaquinone present in all samples, indicating the formation of the characteristic head group fragments of a menaquinone (m/z 187.072) from the exact mass of MK-5 (H2). The insert in the bottom panel shows MS/MS fragmentation spectra of the ubiquinone present only in JS_SRB250Lac^T indicating the formation of the characteristic head group fragments of a ubiquinone (m/z 197.079) from the exact mass of Q-5.

Supplementary Table S1. Genome characteristics of strains IS4^T, JS_SRB250Lac^T and MSL86^T.

Strain	IS4^T	JS_SRB250Lac^T	MSL86^T
Accession	JBSJYP010000000	GCA_016919005.1	GCA_900143695.1
Size (bp)	5,560,733	4,896,277	6,065,581
GC Content (%)	48.1	48.7	49.6
N50	-	625452	174344
L50	1	3	12
Number of Contigs (with PEGs)	1	79	103
Number of Coding Sequences (RAST)	5688	4926	5908
Number of RNAs	62	50	54
Completeness (CheckM2)	97.18	97.62	98.27
Contamination (CheckM2)	3.1	0.59	5.16

Supplementary Table S2. Cellular fatty acid composition of strain IS4^T and its closest phylogenic relative *D. inferna* JS_SRB250Lac^T in comparison to *D. aestuarii* MSL86^T (3) representing the type species of the genus *Desulfopila*. Numbers depicted in the table are percentages of total fatty acids. Major cellular fatty acids (> 5%) of individual strains are indicated in bold. All strains were grown with lactate and sulfate at 28 °C.

	IS4^T	JS_SRB250Lac^T	MSL86^T
Saturated fatty acids			
$C_{14:0}$	1.0	-	2.3
$C_{15:0}$	0.4	-	2.1
$C_{16:0}$	**14.6**	**15.6**	**20.4**
$C_{17:0}$	2.2	**5.0**	1.1
$C_{18:0}$	3.1	**6.9**	3.6
Mono-unsaturated fatty acids			
$C_{14:1}$ ω7c	0.6	-	-
$C_{14:1}$ ω5c	0.5	-	0.8
$C_{15:1}$ ω6c	0.4	1.5	1.7
$C_{16:1}$ ω7c	**18.0**	**25.3**	**18.7**
$C_{16:1}$ ω5c	**24.8**	**13.8**	**31.6**
$C_{17:1}$ ω8c	0.7	-	-
$C_{17:1}$ ω6c	**6.6**	**7.5**	**11.6**
$C_{18:1}$ ω9c	0.5	-	-
$C_{18:1}$ ω7c	**15.2**	**13.1**	2.4
$C_{18:1}$ ω5c	4.6	1.8	2.0
Cyclo fatty acids			
$C_{17:0}$ CYCLO ω7c	1.5	3.5	-
Hydroxy fatty acids			
$C_{14:0}$ 3OH	4.8	3.1	1.6
$C_{16:0}$ 3OH	-	2.0	-

Supplementary Table S3. Polar lipid composition of strains IS4^T, JS_SRB250Lac^T and MSL86^T. The theoretical exact mass of each species and the detected mass of the [M+H]$^+$ ion is shown for each strain.

	Calculated [M+H]$^+$	IS4^T Detected [M+H]$^+$	JS_SRB250Lac^T Detected [M+H]$^+$	MSL86^T Detected [M+H]$^+$
**PE Species**				
31:1	676.4917	-	676.4895	676.4887
31:2	674.4761	-	-	674.4730
32:1	690.5074	690.5042	690.5027	690.5050
32:2	688.4917	688.4890	688.4886	-
33:1	704.5230	704.5197	704.5194	704.5199
33:2	702.5074	702.5046	702.5043	-
34:1	718.5387	718.5356	718.5353	-
34:2	716.5230	716.5201	716.5194	-
35:1	732.5543	-	732.5502	-
35:2	730.5387	730.5346	730.5346	-
36:1	746.5700	746.5671	746.5656	-
36:2	744.5543	744.5509	744.5505	-
37:2	758.5700	758.5672	-	-
**LPE Species**				
16:1	452.2777	-	452.2757	452.2760
18:1	480.3090	-	480.3067	-
**PS Species**				
30:1	706.4659	-	-	706.4629
32:2	732.4816	-	-	732.4788
**PG Species**				
35:1	763.5489	-	763.5449	-
**DPG Species**				
62:3	1319.9018	-	-	1319.8940
62:4	1317.8862	-	-	1317.8804
63:3	1333.9174	-	-	1333.9108
64:2	1349.9488	1349.9427	1349.9429	1349.9398
64:3	1347.9331	1347.9268	-	1347.9266
64:4	1345.9175	-	-	1345.9159
65:2	1363.9644	1363.9575	1363.9595	-
65:3	1361.9488	1361.9426	1361.9429	1361.9420
66:2	1377.9800	1377.9780	1377.9806	-
66:3	1375.9644	1375.9644	1375.9615	-
67:2	1391.9957	1391.9929	1391.9957	-
67:3	1389.9801	1389.9784	1389.9775	-
68:3	1403.9957	1403.9976	1403.9956	-
68:4	1401.9801	1401.9734	-	-
69:3	1418.0114	1418.0109	1418.0110	-
**PC Species**				
32:2	730.5387	730.5346	-	-
33:2	744.5543	744.5550	-	-
34:1	760.5856	760.5828	-	-
34:2	758.5700	758.5707	-	-
36:2	786.6012	786.5984	-	-

LPC Species				
16:0	496.3403	496.3371	-	-
16:1	494.3247	494.3222	-	-
18:1	522.3560	522.3527	-	-
